# Pyrexia as the primary paradox: celiac disease masked as fever of unknown origin

**DOI:** 10.1093/omcr/omag180

**Published:** 2026-10-04

**Authors:** Soufiane Abdouh, Imane Boujguenna, Soukaina Oumlil

**Affiliations:** Primary Care Physician, Marrakesh Medina, 40000, Kingdom of Morocco; Pathologist, Faculty of Medicine and Pharmacy, Guelmim 81000, Kingdom of Morocco; Internal Medicine Specialist, Agadir 81000, Kingdom of Morocco

**Keywords:** celiac disease, fever of unknown origin, Anemia, transglutaminases antibodies, duodenal biopsy, atrophy, gluten-free

## Abstract

Celiac disease (CD) can present solely with atypical extra-intestinal symptoms. We describe a 33-year-old woman hospitalized for a one-month history of fever, myalgias, and tachycardia. Extensive infectious, autoimmune, and malignancy investigations were unrevealing. However, hypochromic anemia and elevated CRP prompted serologic testing, revealing positive anti-transglutaminase IgA. Duodenal biopsy confirmed CD with Marsh IIIa villous atrophy. This case illustrates CD as a rare etiology of fever of unknown origin (FUO), emphasizing the importance of celiac serology in the differential diagnosis of prolonged unexplained fever.

## Introduction

Celiac disease (CD) is an immune-mediated enteropathy triggered by gluten in genetically predisposed individuals, leading to characteristic duodenal villous atrophy [1]. The global seroprevalence is approximately 1.4%, and adults often present with non-classic extraintestinal features such as iron deficiency anemia, that can overshadow gastrointestinal symptoms and delay diagnosis [2, 3]. Fever of unknown origin (FUO) is an exceptionally rare presentation of CD, documented only in isolated case reports, including cases where fever persisted for weeks before serological diagnosis [4, 5]. In this report, we discuss a case of CD presenting as a one-month history of unexplained high-grade fever. Through this report, we aim to enhance clinicians’ awareness regarding this rare cause of FUO.

## Case presentation

A 33-year-old Moroccan female with no significant past medical history presented to the office with a 1-month history of myalgias and documented fever up to 39°C (102.2°F). The patient reported that her symptoms began gradually in the preceding weeks, with diffuse muscle pain accompanied by intermittent fever. She denied gastrointestinal symptoms including abdominal pain, diarrhea, or weight loss. She also denied recent travel, sick contacts, or new medication.

Upon arrival, the patient appeared pale and uncomfortable but in no acute distress. She had thin body habitus. Vital signs were significant for tachycardia (heart rate of 130 bpm), hypotension (blood pressure of 95/55 mmHg). The rest of her physical examination was unremarkable, with no lymphadenopathy, hepatosplenomegaly, or focal neurological deficits. The patient was transferred to an inpatient setting for expedited evaluation.

Laboratory evaluation summarized in Table 1, revealed microcytic hypochromic anemia with a hemoglobin level of 8 g/dL and low ferritin level of 7 ng/mL. Inflammatory markers were elevated, with a C-reactive protein (CRP) level of 140 mg/L and procalcitonin level of 0.33 ng/mL.

**Table 1:** Laboratory evaluation across the clinical timeline.

Lab test	Day 1 Initial Result	Day 10 Discharge	3 months Follow-Up	Normal Range
Hb, g/dL	8.0	8.4	10.2	12–16
MCV, fL	68	70	74	80–100
MCH,pg	20	21	23	27–31
MCHC, g/dL	30	30.5	31	32–36
RDW, %	17%	16.8	16.0	11.5–15
WBC, 10^9^/L	7.6	7.0	6.8	4.0–11.0
Platelets, 10^9^/L	320	310	290	150–400
Ferritin, ng/mL	7	8	12	12–150
CK, U/L	110	105	115	25–200
CRP, mg/L	140	75	5	<3
Procalcitonin, ng/mL	0.33	0.04	-	<0.05
Urinalysis/urine culture	Negative	-	-	-
Blood cultures	Negative	-	-	-
SPE	Normal	-	-	-
HIV Ag/Ab	Nonreactive	-	-	-
HBV (AgHBs)	Nonreactive	-	-	-
HCV Ab	Nonreactive	-	-	-
QuantiFERON-TB Gold Plus	Negative	-	-	-
ANCA titer	Negative	-	-	-
ANA titer	Negative	-	-	-​
tTG-IgA, U/mL	37	-	-	<4

Hb hemoglobin; MCV Mean Corpuscular Volume; MCH Mean Corpuscular Hemoglobin; MCHC Mean Corpuscular Hemoglobin Concentration; RDW Red Cell Distribution Width; WBC White Blood Cell count; CK = creatine kinase; CRP C-reactive protein; SPE Serum Protein Electrophoresis; HBV hepatitis B virus; AgHBs Hepatitis B surface antigen; HCV Ab Hepatitis C virus Antibody; QuantiFERON-TB Gold Plus Interferon-Gamma Release Assay for Tuberculosis; ANCA = Antineutrophil Cytoplasmic Antibody; ANA Antinuclear Antibody; tTG-IgA anti–tissue transglutaminase IgA.

Creatine kinase (CK) levels were within normal limits, and electromyography showed no evidence of myopathy. Infectious workup including blood cultures, urinalysis and urine culture were negative. Serologies for human immunodeficiency virus (HIV), hepatitis B virus (HBV), and hepatitis C virus (HCV) were negative. Transthoracic echocardiography showed no evidence of endocarditis. Tuberculosis workup including QuantiFERON-TB Gold was negative.

Chest Computed tomography (CT), abdomen, and pelvis demonstrated no evidence of malignancy, abscess, or vascular pathology. Serum protein electrophoresis was normal.

Given the prolonged fever and negative infectious and imaging workup, an immunological evaluation was pursued, and antinuclear antibodies (ANA) and antineutrophil cytoplasmic antibodies (ANCA) were negative, anti-tissue transglutaminase IgA antibodies were ordered given the thin habitus, pallor and anemia which returned positive at 37 U/mL.

Based on this finding, an upper endoscopy was performed and showed flattening of duodenal folds. Duodenal biopsy was performed and histopathology showed partial villous atrophy, cryptic hyperplasia, and marked intraepithelial lymphocytes consistent with modified Marsh stage IIIa celiac disease (Fig. 1).

**Figure 1:**
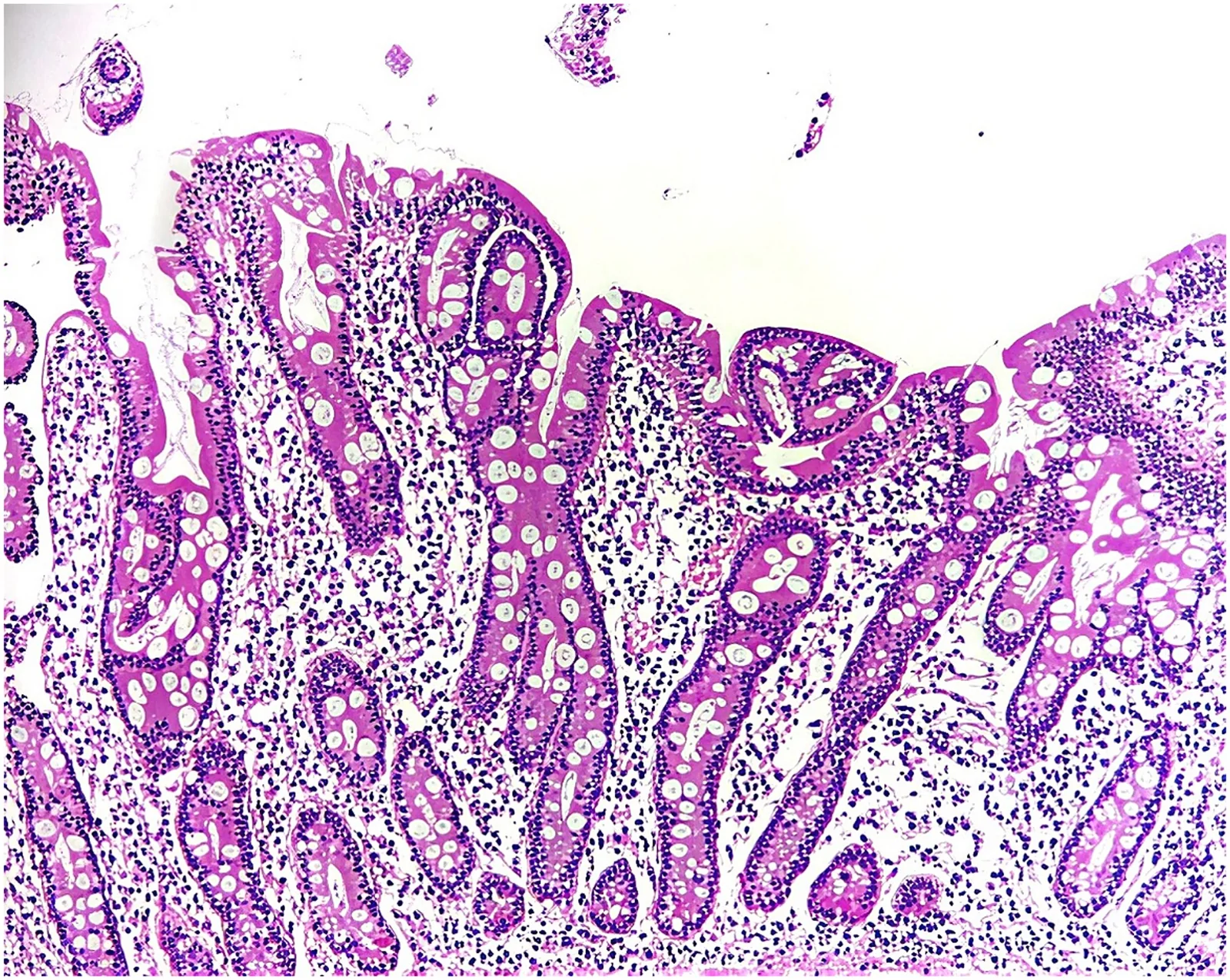
Duodenal biopsy with evidence of villous atrophy and intraepithelial lymphocytes suggesting Marsh stage IIIa celiac disease.

During hospitalization, the patient was managed symptomatically with paracetamol for fever and myalgias and received intravenous iron for treatment of iron-deficiency anemia. Inflammatory markers subsequently normalized by day 10, and she was discharged on a gluten-free diet (GFD). At 3-month follow-up, she reported clinical improvement with resolution of constitutional symptoms, weight gain, and improvement in anemia, consistent with response to dietary therapy and iron repletion in celiac-associated malabsorption (Table 2).

**Table 2:** Clinical course summary.

Timepoint	Clinical status	Key investigations	Management	Outcome
**Day 1: Initial presentation**	1 month of fever and myalgias; tachycardia with hypotension → admitted to inpatient setting	Microcytic anemia (Hb 8 g/dL); tTG-IgA positive; CRP elevated; infectious and malignancy workup negative; upper endoscopy performed	Paracetamol; IV iron	Clinical stabilization
**Day 10: Discharge**	Afebrile; clinically improved	Duodenal biopsy: villous atrophy suggestive of celiac disease; CRP trended down	Gluten-free diet initiated	Discharged home
**Follow-up at 3 months**	Symptom resolution; weight gain	Anemia improved slightly (Hb 10.2 g/dL)	Continued gluten-free diet; oral iron supplementation	Sustained clinical and hematologic improvement

CRP C-reactive protein; tTG-IgA anti–tissue transglutaminase IgA; IV Intravenous.

## Discussion

Celiac disease (CD) is increasingly recognized to present with atypical manifestations, and fever may be the dominant complaint prompting evaluation for fever of unknown origin (FUO) rather than gastrointestinal symptoms. A published case report described CD diagnosed during FUO evaluation in a young female, emphasizing that clinicians should maintain a low threshold to screen for CD when standard infectious, malignant, and inflammatory investigations are unrevealing [4]. Other reports link CD to periodic fever phenotypes and to severe hyperinflammatory syndromes where persistent fever is prominent, reinforcing that immune-driven fever can be part of the CD spectrum [6, 7].

The classic definition of FUO requires fever > 38.3°C on several occasions for at least 3 weeks with no diagnosis after 1 week of inpatient evaluation [8]. Contemporary practice has shifted toward a structured minimal diagnostic evaluation often performed largely as an outpatient before applying the FUO label and before initiating empiric antimicrobials in stable, non-neutropenic patients [9]. In our patient, the 1-month duration satisfies the time criterion, and the negative FUO work-up supports categorizing the presentation as classic FUO prior to identifying CD as the underlying diagnosis [8, 9].

Fever in CD is plausibly mediated by immune activation with systemic release of endogenous pyrogenic cytokines following antigen exposure [10].

In controlled gluten-challenge studies of treated CD, gluten exposure induces a rapid systemic cytokine response, with IL-2 representing one of the earliest and most prominent circulating cytokines following gluten ingestion [11]. This early IL-2 release reflects the reactivation of gluten-specific CD4+ T cells, which are central to the adaptive immune response in CD; more recent evidence further demonstrates that gluten-stimulated IL-2 secretion closely reflects the presence and activity of pathogenic gluten-specific T cells [12]. The ensuing immune response is accompanied by elevations in additional inflammatory mediators, including IL-17A, IFN-γ, IL-6 and TNF-α [11]. This coordinated cytokine response provides a plausible mechanism of fever following gluten exposure. Rapid antigen-specific T-cell activation and IL-2 release may initiate and amplify systemic inflammation, leading to the release of downstream pyrogenic cytokines, particularly IL-6 and TNF-α, which in turn promote prostaglandin E2-mediated signaling within hypothalamic thermoregulatory pathways and thereby increase the body-temperature set point. Thus, fever in CD may represent an uncommon systemic manifestation of acute gluten-driven T-cell activation rather than a direct intestinal effect of gluten alone. This proposed immunologic mechanism is consistent with the clinical course observed in our patient, whose symptoms improved following strict gluten avoidance over a three-month period.

FUO differentials are broad but are classically organized into infectious, malignancies, noninfectious inflammatory diseases, drug fever, and undiagnosed cases [9, 13]. Practical high-yield considerations during initial evaluation typically include tuberculosis and occult abscesses, lymphoma, and systemic inflammatory disorders, while avoiding premature closure once early testing is negative [9, 13].

CD can be an occult yet treatable cause of classic FUO in adults; in a 33-year-old woman with 1 month of fever and negative FUO work-up, positive IgA serology and duodenal biopsy showing villous atrophy appropriately established the diagnosis. Symptomatic improvement after initiation of a GFD strengthens clinicopathologic correlation and aligns with prior FUO-presenting CD reports and with human data demonstrating gluten-triggered systemic cytokine release [4, 11]. In stable FUO, the work-up should remain hypothesis-driven and iterative, while also incorporating diagnoses such as CD when routine pathways fail to yield an explanation.

## Conclusion

Celiac disease can present atypically as an occult cause of classic fever of unknown origin, even in the complete absence of gastrointestinal symptoms. Clinicians should maintain a low threshold for ordering celiac serology when evaluating prolonged unexplained fever, particularly when accompanied by microcytic anemia. Ultimately, recognizing this rare, cytokine-driven inflammatory phenotype ensures timely diagnosis and successful management with a gluten-free diet.

## Supplementary Material

CARE_Check-list_omag180
