# Supplementary material for: Pyrexia as the primary paradox: celiac disease masked as fever of unknown origin
Source: Oxf Med Case Reports. 2026 Oct 4;2026(10):omag180. doi: 10.1093/omcr/omag180 (PMC13634197; doi:10.1093/omcr/omag180)
Supplement: Supplementary file 1 [file care_check-list_omag180.docx]

**CARE Check-list**

- **1. Title:** Pyrexia as the Primary Paradox: Celiac Disease Masked as Fever of Unknown Origin.
- **2. Key Words:** Celiac disease; Fever of Unknown Origin; Anemia; Transglutaminases antibodies; Duodenal biopsy; Atrophy; Gluten-Free.
- **3. Abstract:** The abstract outlines a case of atypical, extra-intestinal celiac disease presenting as classic Fever of Unknown Origin (FUO). It highlights a 33-year-old Moroccan female presenting with a one-month history of high-grade fever, myalgias, and tachycardia, whose extensive standard FUO screening was completely negative. Targeted serology (positive anti-tissue transglutaminase IgA) and subsequent duodenal histopathology confirming modified Marsh stage IIIa villous atrophy successfully established the diagnosis.
- **4. Introduction:** The introduction defines celiac disease as a gluten-induced, immune-mediated enteropathy that often presents with non-classic, extraintestinal manifestations in adulthood. It notes that fever of unknown origin is an exceptionally rare manifestation and outlines the objective to enhance clinical vigilance for this atypical diagnostic presentation.
- **5. Patient Information:** A previously healthy 33-year-old Moroccan female presented with a 1-month history of documented fever up to 39°C (102.2°F) and gradual-onset diffuse myalgias. She denied abdominal pain, diarrhea, weight loss, recent travel, sick contacts, or new medications.
- **6. Clinical Findings:** Physical examination revealed severe pallor, thin body habitus, sinus tachycardia (130 bpm), and hypotension (95/55 mmHg). Laboratory data demonstrated profound microcytic hypochromic anemia (Hb 8 g/dL, Ferritin 7 ng/mL) and marked systemic inflammation (CRP 140 mg/L, Procalcitonin 0.33 ng/mL).
- **7. Timeline:** The timeline tracks the patient from her 1-month outpatient prodrome through her acute inpatient admission, including diagnostic infectious/autoimmune screening, cross-sectional imaging, diagnostic endoscopy on day 4, initialization of supportive therapies, systemic normalization and discharge on day 10, and a comprehensive 3-month follow-up showing total resolution.
- **8. Diagnostic Assessment:** Key diagnostic methods included negative serial blood/urine cultures, unrevealing chest/abdomen/pelvis CT imaging, normal CPK, negative ANA/ANCA panels, positive anti-tissue transglutaminase IgA serology (37 U/mL), and histopathological analysis of a duodenal endoscopic biopsy showing partial villous atrophy and intraepithelial lymphocytes.
- **9. Therapeutic Intervention:** Acute treatment involved symptomatic antipyretic therapy with paracetamol and intravenous iron repletion for iron-deficiency anemia. Long-term causal therapy consisted of strict compliance with a lifelong gluten-free diet (GFD).
- **10. Follow-up and Outcomes:** At the 3-month follow-up assessment, the patient demonstrated complete resolution of constitutional symptoms and pyrexia, substantial weight gain, and correction of her microcytic anemia following diet adherence.
- **11. Discussion:** The discussion highlights the rarity of pyrexia as the dominant complaint of adult celiac disease. It contextualizes classic FUO criteria, details a hypothesis-driven differential diagnostic pathway, and provides an immunobiological explanation where gluten-reactive T-cell activation drives systemic pyrogenic cytokine release (specifically IL-2).
- **12. Patient Perspective:** Following initiation of the gluten-free diet and iron repletion, the patient experienced definitive clinical improvement, full resolution of diffuse muscle pain, and an improved quality of life.
- **13. Informed Consent:** It is explicitly declared in the manuscript text that written informed consent was formally obtained from the patient for the inclusion of all clinical data and biopsy details in this report.
